# Neoadjuvant Versus Adjuvant Chemo-Immunotherapy in Early-Stage Triple-Negative Breast Cancer: A National Cancer Database Study

**DOI:** 10.3390/cancers18213490

**Published:** 2026-10-29

**Authors:** Taiga Komatsu, Rima Patel, Amy Tiersten

**Affiliations:** 1 Department of Internal Medicine, Mount Sinai Morningside/West, 1000 Tenth Avenue, New York, NY 10019, USA; 2 Department of Medicine, Division of Hematology and Medical Oncology, Tisch Cancer Institute, Icahn School of Medicine at Mount Sinai, 1 Gustave L. Levy Place, New York, NY 10029, USA

**Keywords:** triple-negative breast cancer, neoadjuvant therapy, immunotherapy, chemo-immunotherapy, National Cancer Database, overall survival

## Abstract

**Background/Objectives:** While neoadjuvant chemo-immunotherapy has become a standard treatment for patients with early-stage triple-negative breast cancer (TNBC) based on KEYNOTE-522, the trial did not directly compare neoadjuvant with adjuvant chemo-immunotherapy. We compared treatment patterns and survival outcomes between neoadjuvant and adjuvant chemo-immunotherapy in real-world settings. **Methods:** We identified women with clinical stage II–III TNBC diagnosed from 2017–2023 in the National Cancer Database (NCDB) who underwent definitive surgery and received chemo-immunotherapy. Factors associated with neoadjuvant treatment were evaluated. Overall survival (OS) was assessed using Kaplan–Meier curves, univariable and multivariable Cox regression, and propensity-score overlap weighting. Prespecified subgroup analyses were performed by clinical T and N stage. **Results:** Among 21,757 women, 21,340 received neoadjuvant and 417 received adjuvant chemo-immunotherapy. Higher clinical T stage, node-positive disease, and diagnosis in 2021–2023 were associated with neoadjuvant treatment. One-, 2-, and 3-year OS were 98.8%, 93.2%, and 88.6% with neoadjuvant versus 94.3%, 88.9%, and 81.6% with adjuvant chemo-immunotherapy, respectively. Neoadjuvant treatment was associated with improved OS in univariable analysis (HR 0.58, 95% CI 0.46–0.72; *p* < 0.001), multivariable analysis (HR 0.52, 95% CI 0.42–0.66; *p* < 0.001), and after overlap weighting (HR 0.57, 95% CI 0.45–0.73; *p* < 0.001). Associations consistently favored neoadjuvant treatment across clinical T- and N-stage subgroups. **Conclusions:** In this retrospective NCDB study, neoadjuvant chemo-immunotherapy was associated with improved OS compared with adjuvant chemo-immunotherapy. These hypothesis-generating findings are consistent with current clinical practice but should be interpreted cautiously, as differences in treatment regimens and completion rates, which could not be assessed in the NCDB, may have contributed to the observed survival benefit.

## Simple Summary

Neoadjuvant chemo-immunotherapy has become a standard treatment for eligible patients with early-stage triple-negative breast cancer. However, few studies have directly compared neoadjuvant with adjuvant chemo-immunotherapy in this setting. We used the National Cancer Database to compare treatment patterns and overall survival between these treatments in women with clinical stage II–III triple-negative breast cancer who underwent definitive surgery. Among 21,757 women, 21,340 received neoadjuvant and 417 received adjuvant chemo-immunotherapy. Higher clinical T stage, node-positive disease, and later diagnosis year were associated with receipt of neoadjuvant treatment. One-, 2-, and 3-year overall survival were higher with neoadjuvant than adjuvant chemo-immunotherapy. Neoadjuvant treatment was associated with improved overall survival in univariable, multivariable, and propensity-score overlap-weighted analyses, with consistent associations across clinical T- and N-stage subgroups. These hypothesis-generating findings are consistent with current guideline-recommended neoadjuvant chemo-immunotherapy, although the observed survival difference may also reflect other unmeasured treatment-related factors.

## 1. Introduction

Neoadjuvant pembrolizumab combined with chemotherapy followed by adjuvant pembrolizumab has become standard treatment for eligible patients with early-stage triple-negative breast cancer (TNBC) [1,2,3]. In KEYNOTE-522, pembrolizumab was incorporated into the neoadjuvant chemotherapy and continued after surgery, establishing a perioperative treatment strategy that begins while the primary tumor remains in place. The regimen significantly increased pathologic complete response and subsequently demonstrated improvements in event-free survival and overall survival (OS) [4,5,6]. These findings changed the treatment paradigm for early-stage TNBC and led to incorporation of neoadjuvant pembrolizumab-based chemo-immunotherapy followed by adjuvant pembrolizumab into clinical practice guidelines [7].

In contrast, adjuvant-only immune checkpoint inhibitor (ICI) strategies have not demonstrated consistent improvement in their primary efficacy endpoints in TNBC, including randomized trials of adjuvant atezolizumab and avelumab and a recent National Cancer Database (NCDB) analysis of adjuvant chemo-immunotherapy [8,9,10].

Evidence from other malignancies suggests that treatment timing could be important for ICI [11,12]. In melanoma, SWOG S1801 demonstrated improved event-free survival with pembrolizumab administered before and after surgery compared with adjuvant-only pembrolizumab [13]. In resectable non-small cell lung cancer, an NCDB analysis similarly reported improved survival with neoadjuvant chemo-immunotherapy [14]. However, KEYNOTE-522 did not directly compare neoadjuvant with adjuvant chemo-immunotherapy, and direct comparative data on these treatment cohorts remain limited in TNBC.

We therefore used the NCDB to evaluate factors associated with receipt of neoadjuvant treatment and to compare OS between neoadjuvant and adjuvant chemo-immunotherapy in women with clinical stage II–III TNBC.

## 2. Materials and Methods

### 2.1. Data Source and Ethical Considerations

We used the 2024 NCDB Participant User File. The NCDB is a hospital-based oncology database containing de-identified data from Commission on Cancer-accredited facilities across the United States [15,16,17].

The 2017–2023 study period was selected to span the era of early clinical investigation of ICI-based neoadjuvant and adjuvant therapy in TNBC through the subsequent widespread adoption of the KEYNOTE-522 regimen [18,19,20].

The study was reviewed by the Institutional Review Board of the Icahn School of Medicine at Mount Sinai (STUDY-26-01079), which determined that the study did not constitute research involving human subjects as defined by U.S. Department of Health and Human Services and U.S. Food and Drug Administration regulations.

### 2.2. Patient Selection

Women aged ≥ 18 years with clinical stage II–III TNBC diagnosed between 2017 and 2023 who underwent definitive breast surgery and received chemo-immunotherapy were included. Clinical stage II–III disease was identified using the AJCC 7th Edition clinical anatomical stage group (TNM_CLIN_STAGE_GROUP) for patients diagnosed in 2017 and the AJCC 8th Edition clinical prognostic stage group (AJCC_TNM_CLIN_STG_GRP) for those diagnosed between 2018 and 2023. Clinical T, N, and M categories were obtained from the corresponding edition-specific variables and harmonized into common categories for analysis.

Patients were classified according to the timing of initiation of both chemotherapy and immunotherapy relative to definitive surgery. The neoadjuvant chemo-immunotherapy group included patients in whom both treatments were initiated within 365 days before surgery, whereas the adjuvant chemo-immunotherapy group included patients in whom both treatments were initiated within 180 days after surgery. Patients with discordant sequencing, in whom chemotherapy and immunotherapy were initiated on opposite sides of surgery, were excluded. In the neoadjuvant group, whether immunotherapy was continued after surgery could not be determined based on the available information from the NCDB. Furthermore, the NCDB does not provide sufficient information to determine specific systemic treatment regimens, cumulative doses, or completion of the intended treatment course in either group. The NCDB variables and corresponding coding criteria used for patient selection and treatment classification are provided in Supplementary Table S1. The patient selection process and sequential exclusions are illustrated in Supplementary Figure S1.

### 2.3. Statistical Analysis

Patient characteristics were compared using the Pearson χ^2^ test for categorical variables and the Welch two-sample *t* test for continuous variables. Logistic regression was used to evaluate factors associated with receipt of neoadjuvant chemo-immunotherapy. The multivariable model included age, race/ethnicity, insurance status, Charlson–Deyo comorbidity score, clinical T stage, clinical N stage, clinical grade, histology, diagnosis year, facility type, and urban/rural residence. Patients with missing information required for eligibility determination or treatment-sequence classification, including clinical staging and treatment initiation dates, were excluded. Patients with missing survival time or vital status were excluded from the survival analyses. Missing or unknown values for other categorical covariates were retained in separate or combined “Unknown” categories, as appropriate.

Median follow-up was estimated using the reverse Kaplan–Meier method for the overall cohort and separately for each treatment group. One-, 2-, and 3-year OS probabilities with corresponding 95% confidence intervals were estimated using the Kaplan–Meier method. OS was further evaluated using Kaplan–Meier curves with the log-rank test and univariable Cox proportional-hazards regression. Multivariable Cox regression adjusted for the same prespecified demographic, clinical, tumor, diagnosis-year, and facility characteristics. The proportional hazards assumption was assessed using scaled Schoenfeld residuals. Variables violating the assumption were entered as stratifying factors rather than covariates in an additional multivariable Cox model.

Propensity-score overlap weighting was additionally performed using prespecified baseline covariates [21,22]. Covariate balance was assessed using standardized mean differences (SMDs), and weighted Cox regression was performed using robust standard errors.

Prespecified subgroup analyses were performed according to clinical T stage (cT1 vs. cT2-4) and clinical N stage (cN0 vs. cN1-3). Formal interaction tests were conducted by including treatment-by-subgroup interaction terms in multivariable Cox regression models. To assess potential selection and guarantee-time biases, we performed sensitivity analyses. First, we expanded the cohort to include patients who received chemo-immunotherapy but did not undergo surgery, assigning these patients to the neoadjuvant-first group. Second, we performed landmark analyses at 180 and 270 days after diagnosis in the expanded cohort. Patients were included if they were alive and under observation at the landmark and had initiated both chemotherapy and immunotherapy by that time. OS was measured from each landmark, and univariable and multivariable Cox regression analyses were performed using the same baseline covariates as in the primary analysis. Additional sensitivity analyses were performed by restricting the primary cohort to patients diagnosed between 2021 and 2023 and to those with ductal histology, respectively.

All statistical tests were two-sided, and *p* < 0.05 was considered statistically significant. Statistical analyses were performed using R version 4.6.0 (R Foundation for Statistical Computing, Vienna, Austria).

## 3. Results

### 3.1. Patient Characteristics

Among 21,757 women, 21,340 (98.1%) received neoadjuvant and 417 (1.9%) received adjuvant chemo-immunotherapy (Table 1). Patients receiving neoadjuvant treatment were younger than those receiving adjuvant treatment (mean age, 53.8 vs 61.2 years), and several clinical, tumor, and treatment-setting characteristics differed between the groups, as detailed in Table 1.

Use of neoadjuvant chemo-immunotherapy increased over time. Patients diagnosed in 2022–2023 accounted for 72.4% of the neoadjuvant group compared with 49.6% of the adjuvant group, whereas diagnoses in 2017–2020 accounted for 8.8% and 30.2%, respectively.

### 3.2. Factors Associated with Neoadjuvant Treatment

Higher clinical T stage, node-positive disease, and later diagnosis year were associated with neoadjuvant treatment (Table 2). Compared with cT1 disease, adjusted ORs were 1.85 (95% CI 1.28–2.67) for cT2, 2.59 (95% CI 1.67–4.04) for cT3, and 2.54 (95% CI 1.47–4.37) for cT4 disease. Compared with cN0 disease, adjusted ORs were 2.26 (95% CI 1.75–2.92) for cN1 and 3.29 (95% CI 2.04–5.31) for cN2-3 disease. Diagnosis in 2021, 2022, and 2023 was associated with greater odds of neoadjuvant treatment compared with 2017, with adjusted ORs of 2.14 (95% CI 1.15–3.96), 3.36 (95% CI 1.82–6.20), and 3.91 (95% CI 2.11–7.26), respectively.

### 3.3. Overall Survival

The median follow-up was 32.8 months overall (95% CI, 32.6–33.0), 32.7 months in the neoadjuvant group (95% CI, 32.5–33.0), and 38.7 months in the adjuvant group (95% CI, 35.3–41.8). A total of 2259 deaths occurred. There were 2177 deaths among 21,340 women receiving neoadjuvant treatment (10.2%) and 82 among 417 receiving adjuvant treatment (19.7%). Median OS was not reached in either group.

**Figure 1:**
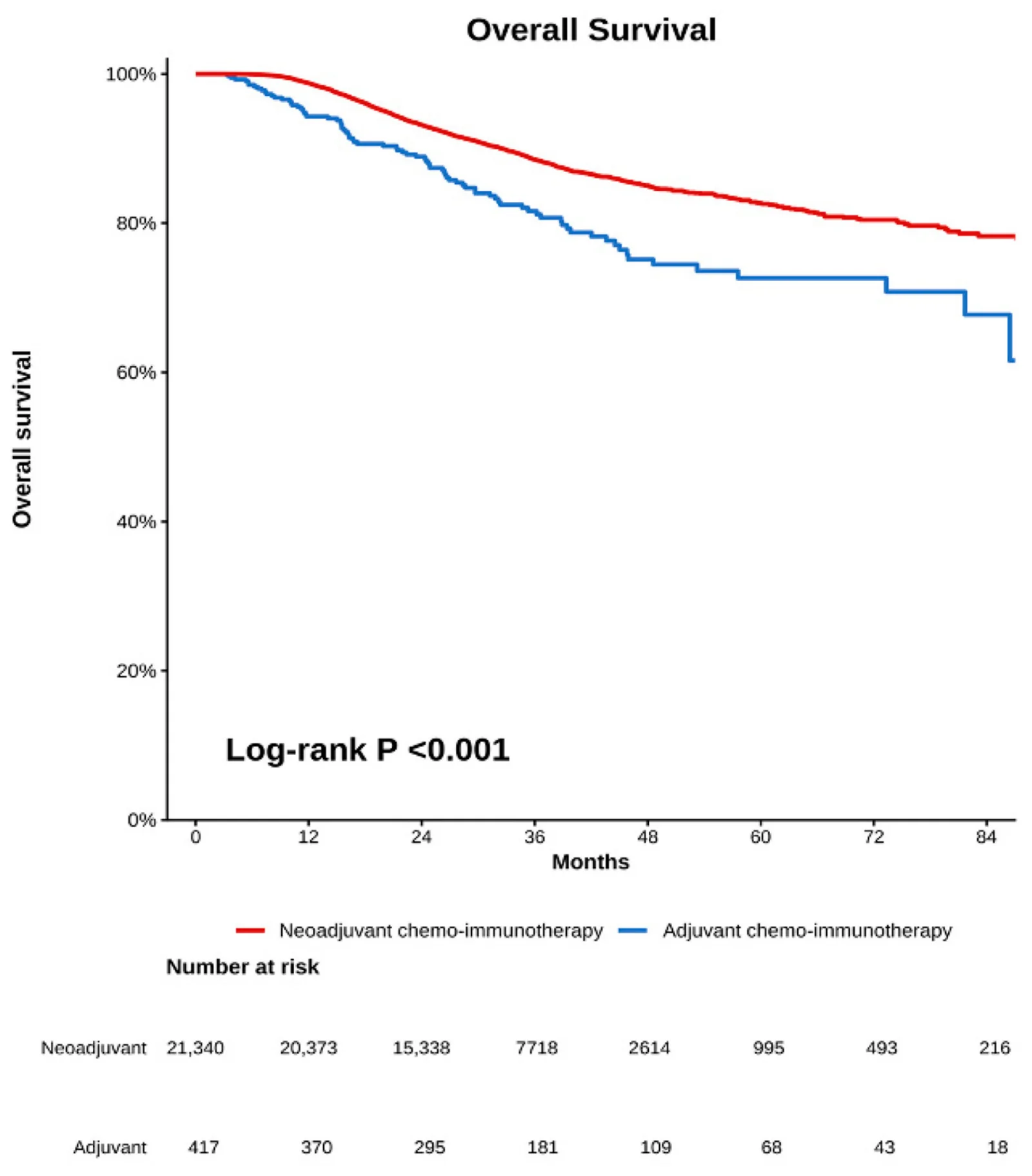
Kaplan–Meier curves of overall survival in neoadjuvant versus adjuvant chemo-immunotherapy.

One-, 2-, and 3-year OS estimates were 98.8% (95% CI, 98.6–98.9%), 93.2% (95% CI, 92.8–93.5%), and 88.6% (95% CI, 88.1–89.1%) with neoadjuvant chemo-immunotherapy, compared with 94.3% (95% CI, 92.1–96.6%), 88.9% (95% CI, 85.8–92.1%), and 81.6% (95% CI, 77.6–85.9%) with adjuvant chemo-immunotherapy, respectively. Kaplan–Meier analysis demonstrated significantly higher OS with neoadjuvant treatment (log-rank *p* < 0.001; Figure 1).

In univariable Cox regression, neoadjuvant chemo-immunotherapy was associated with lower mortality compared with adjuvant treatment (HR 0.58, 95% CI 0.46–0.72; *p* < 0.001). The association remained significant after adjustment for demographic, clinical, tumor, diagnosis-year, and facility characteristics (HR 0.52, 95% CI 0.42–0.66; *p* < 0.001) (Table 3). The proportional hazards assumption was violated for clinical T stage (*p* < 0.001) and histology (*p* = 0.007) in the initial multivariable Cox model. Therefore, clinical T stage and histology were included as stratification factors in an additional multivariable Cox model. In the stratified model, neoadjuvant chemo-immunotherapy remained associated with improved OS (adjusted HR, 0.52; 95% CI, 0.42–0.66; *p* < 0.001).

### 3.4. Propensity-Score Overlap Weighting

Before overlap weighting, the maximum absolute SMD among propensity-score model covariates was 0.525. After weighting, the maximum absolute SMD was <0.001. Formal propensity-model balance is presented in Supplementary Table S2, and covariate balance and propensity-score distributions in Supplementary Figures S2 and S3, respectively. Propensity-score distributions showed overall overlap between the treatment groups, although overlap was reduced at lower propensity scores. The effective sample sizes after overlap weighting were 6331.6 and 413.3 for the neoadjuvant and adjuvant groups, respectively.

After overlap weighting, neoadjuvant chemo-immunotherapy remained associated with improved OS (HR 0.57, 95% CI 0.45–0.73; *p* < 0.001).

### 3.5. Subgroup Analyses

The association consistently favored neoadjuvant chemo-immunotherapy across clinical T- and N-stage subgroups (Table 4). For cT1 disease, the univariable HR was 0.42 (95% CI 0.23–0.78; *p* = 0.006) and the multivariable HR was 0.35 (95% CI 0.19–0.66; *p* < 0.001). For cT2-4 disease, the corresponding HRs were 0.60 (95% CI 0.47–0.76; *p* < 0.001) and 0.57 (95% CI 0.44–0.73; *p* < 0.001). For cN0 disease, the univariable HR was 0.43 (95% CI 0.32–0.59; *p* < 0.001) and the multivariable HR was 0.50 (95% CI 0.37–0.69; *p* < 0.001). For cN1-3 disease, the corresponding HRs were 0.53 (95% CI 0.39–0.73; *p* < 0.001) and 0.56 (95% CI 0.40–0.77; *p* < 0.001). Formal interaction tests did not identify statistically significant differences in the association between treatment sequence and OS according to clinical T stage (*p* for interaction = 0.158) or clinical N stage (*p* for interaction = 0.645).

### 3.6. Sensitivity Analyses

To assess potential selection bias, we expanded the cohort to include 1306 patients who received chemo-immunotherapy but did not undergo surgery. In this expanded cohort (n = 23,063), the association between neoadjuvant treatment and improved OS remained statistically significant (adjusted HR, 0.66; 95% CI, 0.52–0.82). In landmark analyses of the expanded cohort, neoadjuvant-first treatment remained associated with improved OS at both 180 days (adjusted HR, 0.64; 95% CI, 0.50–0.81) and 270 days (adjusted HR, 0.64; 95% CI, 0.50–0.82). The association between neoadjuvant chemo-immunotherapy and improved OS remained consistent in additional sensitivity analyses restricted to patients diagnosed between 2021 and 2023 (n = 19,744; adjusted HR, 0.45; 95% CI, 0.34–0.61) and those with ductal histology (n = 20,318; adjusted HR, 0.46; 95% CI, 0.36–0.59).

## 4. Discussion

In this NCDB study of women with clinical stage II–III TNBC receiving chemo-immunotherapy, higher clinical T stage, node-positive disease, and later diagnosis year were associated with neoadjuvant treatment. OS estimates at 1, 2, and 3 years were consistently higher among patients receiving neoadjuvant chemo-immunotherapy. The association between neoadjuvant chemo-immunotherapy and improved OS remained significant in univariable, multivariable, and propensity-score overlap-weighted analyses and was consistently observed across clinical T- and N-stage subgroups. Despite violations of the proportional hazards assumption for clinical T stage and histology, the treatment association remained largely unchanged in the stratified Cox model, supporting the robustness of the findings. Nevertheless, differences in treatment regimens and completion rates between the groups may also have contributed to the observed survival benefit.

KEYNOTE-522 established neoadjuvant pembrolizumab combined with chemotherapy followed by adjuvant pembrolizumab as standard treatment for eligible patients with stage II–III TNBC after demonstrating improvements in pathologic complete response, event-free survival, and OS [4,5,6,7]. Use of immunotherapy in the neoadjuvant setting has subsequently increased substantially. An NCDB analysis reported that immunotherapy use among patients with early-stage TNBC receiving neoadjuvant therapy increased from 4.2% in 2017 to 48.0% in 2021 [23]. The increasing use of neoadjuvant chemo-immunotherapy across diagnosis years in our cohort, particularly after 2020, is consistent with this change in clinical practice. The associations between higher clinical T and N stage and neoadjuvant treatment also suggest greater use of preoperative systemic therapy among patients with clinically higher-risk disease.

Studies of adjuvant ICI therapy have not shown significant improvement in their primary survival endpoints. ALEXANDRA/IMpassion030 found no significant improvement in invasive disease-free survival with adjuvant atezolizumab plus chemotherapy compared with chemotherapy alone [8]. A-BRAVE did not meet its primary disease-free survival endpoints with adjuvant avelumab, although a descriptive OS analysis favored avelumab [9]. An NCDB study of patients with stage II–III TNBC undergoing surgery similarly reported no significant OS improvement with adjuvant chemotherapy plus immunotherapy compared with chemotherapy alone [10].

Randomized trials in melanoma have demonstrated that initiating ICI before surgery can improve outcomes compared with an adjuvant-only approach [24]. In SWOG S1801, pembrolizumab administered before and after surgery improved event-free survival compared with adjuvant-only pembrolizumab in resectable melanoma [13]. NADINA subsequently demonstrated improved event-free survival with neoadjuvant nivolumab plus ipilimumab compared with adjuvant nivolumab in resectable stage III melanoma [25]. In resectable non-small cell lung cancer, an NCDB analysis also reported improved OS with neoadjuvant compared with adjuvant chemo-immunotherapy [14]. However, KEYNOTE-522 did not directly compare neoadjuvant with adjuvant chemo-immunotherapy in TNBC. The present study therefore addresses a clinically relevant treatment-sequencing question not directly evaluated in the pivotal TNBC trial.

There is biologic plausibility for greater activity when ICI is administered while the primary tumor remains in place [26]. In preclinical breast cancer models, neoadjuvant immunotherapy generated greater systemic antitumor activity and expansion of tumor-specific CD8-positive T cells than the same treatment administered after tumor resection [27]. The intact primary tumor may provide a persistent source of tumor antigens, while tumor-draining lymph nodes may facilitate antigen presentation, T-cell priming, and expansion of tumor-specific clones during checkpoint blockade [28]. In melanoma, OpACIN similarly demonstrated stronger immune activation with neoadjuvant compared with adjuvant checkpoint inhibition [29]. These observations provide a potential biologic explanation for why ICI administered before surgery may differ from postoperative-only treatment. However, the NCDB cannot evaluate immune activation, antigen-specific responses, or other biologic mediators, and these mechanisms should therefore be considered contextual rather than explanatory evidence for the association observed in this study.

Several limitations should be considered. First, the retrospective observational design is subject to treatment-selection bias and residual or unmeasured confounding despite multivariable adjustment and overlap weighting. Second, the adjuvant cohort was substantially smaller than the neoadjuvant cohort, reflecting the uncommon use of this treatment sequence. Markedly unequal treatment groups have also been evaluated in previous NCDB studies of uncommon treatment strategies [10,30]. Importantly, the direction and magnitude of the association in the present study were similar across univariable, multivariable, and overlap-weighted analyses, and overlap weighting achieved excellent balance across measured covariates. Nevertheless, the small comparator group and the possibility of unmeasured differences remain important limitations. Third, the NCDB does not identify the specific immunotherapy agent, chemotherapy regimen, treatment duration, number of doses, treatment completion, reason for treatment selection, performance status, or several other clinical factors that may influence both treatment sequence and outcomes. Therefore, receipt of the complete KEYNOTE-522 regimen, including postoperative continuation of immunotherapy, could not be confirmed in the neoadjuvant group. Furthermore, differences in systemic therapy regimens and treatment completion between groups may have influenced survival outcomes independently of treatment sequencing. Fourth, recurrence, event-free survival, and breast cancer-specific survival are unavailable, leaving OS as the principal survival endpoint. Fifth, defining treatment groups based on subsequent treatment delivery while measuring OS from diagnosis may introduce selection and guarantee-time biases. Although sensitivity analyses including patients without surgery, landmark analyses, and analyses restricted to the more contemporary treatment era and ductal histology yielded consistent associations, these analyses could not fully eliminate potential biases. In particular, the NCDB does not capture the reasons for not undergoing surgery or the intended treatment strategy among patients without surgery [31]. Finally, follow-up was relatively short for early-stage breast cancer, particularly among patients diagnosed in later years.

## 5. Conclusions

In this retrospective NCDB study of patients with clinical stage II–III TNBC, neoadjuvant chemo-immunotherapy was associated with improved OS compared with adjuvant chemo-immunotherapy. The association remained significant after multivariable adjustment and propensity-score overlap weighting and was consistently observed across clinical T- and N-stage subgroups. These hypothesis-generating findings are consistent with current guideline-recommended practice, although the observed survival difference cannot be attributed solely to treatment timing.

## Figures and Tables

**Table 1:** Patient characteristics.

Variable	Category	Neoadjuvant Chemo-Immunotherapy	Adjuvant Chemo-Immunotherapy	*p* Value
Patients		21,340	417	
Age, years		53.8 (13.3)	61.2 (14.9)	<0.001
Race/ethnicity	Non-Hispanic White	12,610 (59.1%)	267 (64.0%)	0.249
	Non-Hispanic Black	4606 (21.6%)	81 (19.4%)	
	Non-Hispanic Asian/Pacific Islander	1099 (5.1%)	22 (5.3%)	
	Hispanic	2317 (10.9%)	34 (8.2%)	
	Other/Unknown	708 (3.3%)	13 (3.1%)	
Insurance	Private	12,593 (59.0%)	175 (42.0%)	<0.001
	Medicare	4988 (23.4%)	187 (44.8%)	
	Medicaid	2760 (12.9%)	33 (7.9%)	
	Uninsured	455 (2.1%)	12 (2.9%)	
	Other/Unknown	544 (2.5%)	10 (2.4%)	
Charlson–Deyo comorbidity score	0	17,827 (83.5%)	320 (76.7%)	<0.001
	1	2555 (12.0%)	67 (16.1%)	
	≥2	958 (4.5%)	30 (7.2%)	
Clinical T stage	cT1	1799 (8.4%)	45 (10.8%)	0.001
	cT2	14,477 (67.8%)	307 (73.6%)	
	cT3	3509 (16.4%)	45 (10.8%)	
	cT4	1555 (7.3%)	20 (4.8%)	
Clinical N stage	cN0	10,899 (51.1%)	288 (69.1%)	<0.001
	cN1	8131 (38.1%)	110 (26.4%)	
	cN2-3	2310 (10.8%)	19 (4.6%)	
Clinical grade	Grade 1–2	3158 (14.8%)	86 (20.6%)	<0.001
	Grade 3	17,026 (79.8%)	262 (62.8%)	
	Unknown	1156 (5.4%)	69 (16.5%)	
Histology	Ductal	19,974 (93.6%)	344 (82.5%)	<0.001
	Metaplastic	483 (2.3%)	41 (9.8%)	
	Other	883 (4.1%)	32 (7.7%)	
Diagnosis year	2017	188 (0.9%)	17 (4.1%)	<0.001
	2018	348 (1.6%)	41 (9.8%)	
	2019	408 (1.9%)	34 (8.2%)	
	2020	943 (4.4%)	34 (8.2%)	
	2021	3995 (18.7%)	84 (20.1%)	
	2022	7558 (35.4%)	107 (25.7%)	
	2023	7900 (37.0%)	100 (24.0%)	
Facility type	Community Cancer Program	562 (2.6%)	30 (7.2%)	<0.001
	Comprehensive Community Cancer Program	5442 (25.5%)	118 (28.3%)	
	Academic/Research Program	6030 (28.3%)	109 (26.1%)	
	Integrated Network Cancer Program	5726 (26.8%)	120 (28.8%)	
	Unknown	3580 (16.8%)	40 (9.6%)	
Urban/rural residence	Metro	18,356 (86.0%)	341 (81.8%)	0.041
	Urban	2244 (10.5%)	59 (14.1%)	
	Rural/Unknown	740 (3.5%)	17 (4.1%)	

**Table 2:** Factors associated with receipt of neoadjuvant versus adjuvant chemo-immunotherapy.

Variable	Category	Univariable OR (95% CI)	*p* Value	Multivariable OR (95% CI)	*p* Value
Age group	Age < 50	Reference		Reference	
	Age 50–70	0.68 (0.53–0.87)	0.002	0.82 (0.60–1.12)	0.212
	Age > 70	0.23 (0.17–0.30)	<0.001	0.34 (0.23–0.51)	<0.001
Race/ethnicity	Non-Hispanic White	Reference		Reference	
	Non-Hispanic Black	1.20 (0.93–1.54)	0.155	0.90 (0.69–1.16)	0.416
	Non-Hispanic Asian/Pacific Islander	1.04 (0.67–1.60)	0.871	0.81 (0.52–1.28)	0.369
	Hispanic	1.42 (1.00–2.04)	0.052	0.98 (0.67–1.43)	0.916
	Other/Unknown	1.11 (0.64–1.93)	0.703	0.88 (0.50–1.54)	0.656
Insurance	Private	Reference		Reference	
	Medicare	0.37 (0.30–0.46)	<0.001	0.65 (0.49–0.87)	0.003
	Medicaid	1.15 (0.79–1.67)	0.466	0.99 (0.68–1.45)	0.966
	Uninsured	0.51 (0.28–0.91)	0.022	0.45 (0.25–0.83)	0.011
	Other/Unknown	0.72 (0.39–1.36)	0.312	0.93 (0.49–1.78)	0.836
Charlson–Deyo comorbidity score	0	Reference		Reference	
	1	0.68 (0.52–0.89)	0.005	0.82 (0.62–1.08)	0.160
	≥2	0.56 (0.39–0.82)	0.003	0.75 (0.51–1.11)	0.156
Clinical T stage	cT1	Reference		Reference	
	cT2	1.19 (0.87–1.63)	0.278	1.85 (1.28–2.67)	<0.001
	cT3	1.95 (1.29–2.95)	0.002	2.59 (1.67–4.04)	<0.001
	cT4	1.92 (1.13–3.25)	0.015	2.54 (1.47–4.37)	<0.001
Clinical N stage	cN0	Reference		Reference	
	cN1	1.95 (1.56–2.43)	<0.001	2.26 (1.75–2.92)	<0.001
	cN2-3	3.14 (1.98–4.97)	<0.001	3.29 (2.04–5.31)	<0.001
Clinical grade	Grade 1–2	Reference		Reference	
	Grade 3	1.78 (1.39–2.27)	<0.001	1.41 (1.09–1.82)	0.009
	Unknown	0.46 (0.33–0.63)	<0.001	0.48 (0.33–0.69)	<0.001
Histology	Ductal	Reference		Reference	
	Metaplastic	0.20 (0.14–0.28)	<0.001	0.27 (0.19–0.39)	<0.001
	Other	0.47 (0.33–0.68)	<0.001	0.53 (0.36–0.78)	0.001
Diagnosis year	2017	Reference		Reference	
	2018	0.78 (0.43–1.40)	0.406	0.36 (0.18–0.70)	0.003
	2019	1.10 (0.60–2.01)	0.758	0.47 (0.24–0.93)	0.031
	2020	2.54 (1.40–4.61)	0.002	1.15 (0.59–2.26)	0.684
	2021	4.39 (2.57–7.50)	<0.001	2.14 (1.15–3.96)	0.016
	2022	6.53 (3.86–11.05)	<0.001	3.36 (1.82–6.20)	<0.001
	2023	7.30 (4.30–12.39)	<0.001	3.91 (2.11–7.26)	<0.001
Facility type	Community Cancer Program	Reference		Reference	
	Comprehensive Community Cancer Program	2.49 (1.66–3.74)	<0.001	2.30 (1.50–3.51)	<0.001
	Academic/Research Program	2.99 (1.98–4.50)	<0.001	2.92 (1.89–4.50)	<0.001
	Integrated Network Cancer Program	2.58 (1.72–3.87)	<0.001	2.48 (1.62–3.81)	<0.001
	Unknown	4.79 (2.97–7.74)	<0.001	2.42 (1.40–4.20)	0.002
Urban/rural residence	Metro	Reference		Reference	
	Urban	0.70 (0.53–0.93)	0.013	0.73 (0.54–0.98)	0.038
	Rural/Unknown	0.79 (0.48–1.28)	0.335	0.87 (0.52–1.43)	0.572

Abbreviations: OR, odds ratio; CI, confidence interval.

**Table 3:** Univariable and multivariable Cox regression analyses for overall survival.

Variable		Unadjusted HR (95% CI)	*p* Value	Adjusted HR (95% CI)	*p* Value
Chemo-immunotherapy sequence	Neoadjuvant chemo-immunotherapy	0.58 (0.46–0.72)	<0.001	0.52 (0.42–0.66)	<0.001
	Adjuvant chemo-immunotherapy	Reference		Reference	
Age group	Age < 50	Reference		Reference	
	Age 50–70	1.17 (1.06–1.28)	<0.001	1.13 (1.00–1.27)	0.041
	Age > 70	1.85 (1.63–2.09)	<0.001	1.45 (1.21–1.72)	<0.001
Race/ethnicity	Non-Hispanic White	Reference		Reference	
	Non-Hispanic Black	1.25 (1.13–1.38)	<0.001	1.17 (1.06–1.30)	0.002
	Non-Hispanic Asian/Pacific Islander	0.68 (0.55–0.86)	<0.001	0.75 (0.60–0.94)	0.012
	Hispanic	0.84 (0.73–0.98)	0.027	0.83 (0.71–0.98)	0.024
	Other/Unknown	1.00 (0.79–1.27)	0.987	1.03 (0.81–1.31)	0.780
Insurance	Private	Reference		Reference	
	Medicare	1.59 (1.45–1.75)	<0.001	1.26 (1.11–1.42)	<0.001
	Medicaid	1.32 (1.16–1.49)	<0.001	1.13 (0.99–1.28)	0.061
	Uninsured	1.12 (0.83–1.51)	0.448	1.00 (0.74–1.35)	0.992
	Other/Unknown	1.03 (0.78–1.36)	0.837	0.96 (0.73–1.28)	0.793
Charlson–Deyo comorbidity score	0	Reference		Reference	
	1	1.28 (1.13–1.44)	<0.001	1.19 (1.05–1.34)	0.005
	≥2	1.74 (1.47–2.05)	<0.001	1.52 (1.29–1.80)	<0.001
Clinical T stage	cT1	Reference		Reference	
	cT2	0.83 (0.71–0.97)	0.022	1.31 (1.11–1.54)	0.001
	cT3	1.74 (1.47–2.06)	<0.001	2.27 (1.91–2.70)	<0.001
	cT4	2.99 (2.50–3.57)	<0.001	3.04 (2.53–3.66)	<0.001
Clinical N stage	cN0	Reference		Reference	
	cN1	2.20 (2.00–2.42)	<0.001	2.15 (1.94–2.37)	<0.001
	cN2-3	3.81 (3.39–4.28)	<0.001	3.13 (2.76–3.55)	<0.001
Clinical grade	Grade 1–2	Reference		Reference	
	Grade 3	1.04 (0.92–1.17)	0.506	1.09 (0.96–1.23)	0.170
	Unknown	1.18 (0.98–1.42)	0.081	1.05 (0.85–1.30)	0.630
Histology	Ductal	Reference		Reference	
	Metaplastic	1.91 (1.56–2.35)	<0.001	2.03 (1.64–2.50)	<0.001
	Other	1.30 (1.08–1.56)	0.007	0.97 (0.80–1.18)	0.783
Diagnosis year	2017	Reference		Reference	
	2018	0.62 (0.43–0.89)	0.010	0.70 (0.46–1.04)	0.078
	2019	0.89 (0.62–1.26)	0.498	1.01 (0.69–1.50)	0.943
	2020	0.85 (0.62–1.17)	0.326	0.93 (0.64–1.34)	0.681
	2021	0.85 (0.63–1.15)	0.297	0.96 (0.68–1.35)	0.796
	2022	0.84 (0.62–1.13)	0.242	0.96 (0.68–1.36)	0.830
	2023	0.78 (0.57–1.05)	0.102	0.89 (0.63–1.27)	0.533
Facility type	Community Cancer Program	Reference		Reference	
	Comprehensive Community Cancer Program	1.05 (0.81–1.36)	0.704	1.09 (0.84–1.41)	0.503
	Academic/Research Program	0.85 (0.66–1.10)	0.217	0.93 (0.72–1.21)	0.587
	Integrated Network Cancer Program	0.98 (0.76–1.27)	0.898	1.05 (0.81–1.35)	0.732
	Unknown	0.82 (0.63–1.08)	0.155	1.05 (0.79–1.39)	0.734
Urban/rural residence	Metro	Reference		Reference	
	Urban	1.15 (1.01–1.31)	0.031	1.11 (0.98–1.27)	0.105
	Rural/Unknown	0.97 (0.78–1.22)	0.811	1.04 (0.83–1.30)	0.741

Abbreviations: HR, hazard ratio; CI, confidence interval.

**Table 4:** Subgroup analyses of overall survival by clinical T and N stage.

	Subgroup	Unadjusted HR (95% CI)	*p* Value	Adjusted HR (95% CI)	*p* Value
Clinical T stage	cT1	0.42 (0.23–0.78)	0.006	0.35 (0.19–0.66)	<0.001
	cT2-4	0.60 (0.47–0.76)	<0.001	0.57 (0.44–0.73)	<0.001
Clinical N stage	cN0	0.43 (0.32–0.59)	<0.001	0.50 (0.37–0.69)	<0.001
	cN1-3	0.53 (0.39–0.73)	<0.001	0.56 (0.40–0.77)	<0.001

Abbreviations: HR, hazard ratio; CI, confidence interval.

## Data Availability

The National Cancer Database Participant User File is not publicly redistributable and is subject to the applicable American College of Surgeons data-use agreement. Eligible investigators may request access through the American College of Surgeons. Analytic code may be made available by the authors upon reasonable request, subject to applicable data-use restrictions.

## Supplementary Materials

The following supporting information can be downloaded at https://www.mdpi.com/article/10.3390/cancers18213490/s1: Table S1: National Cancer Database variables and coding criteria used for patient selection and treatment classification; Table S2: Propensity-score model covariate balance before and after overlap weighting; Figure S1: Flow diagram of patient selection; Figure S2: Covariate balance before and after propensity-score overlap weighting; Figure S3: Propensity-score distributions.
