# Supplementary material for: Neoadjuvant Versus Adjuvant Chemo-Immunotherapy in Early-Stage Triple-Negative Breast Cancer: A National Cancer Database Study
Source: Cancers (Basel). 2026 Oct 29;18(21):3490. doi: 10.3390/cancers18213490 (PMC13761846; doi:10.3390/cancers18213490)

Supplementary Figure S1. Flow diagram of patient selection.

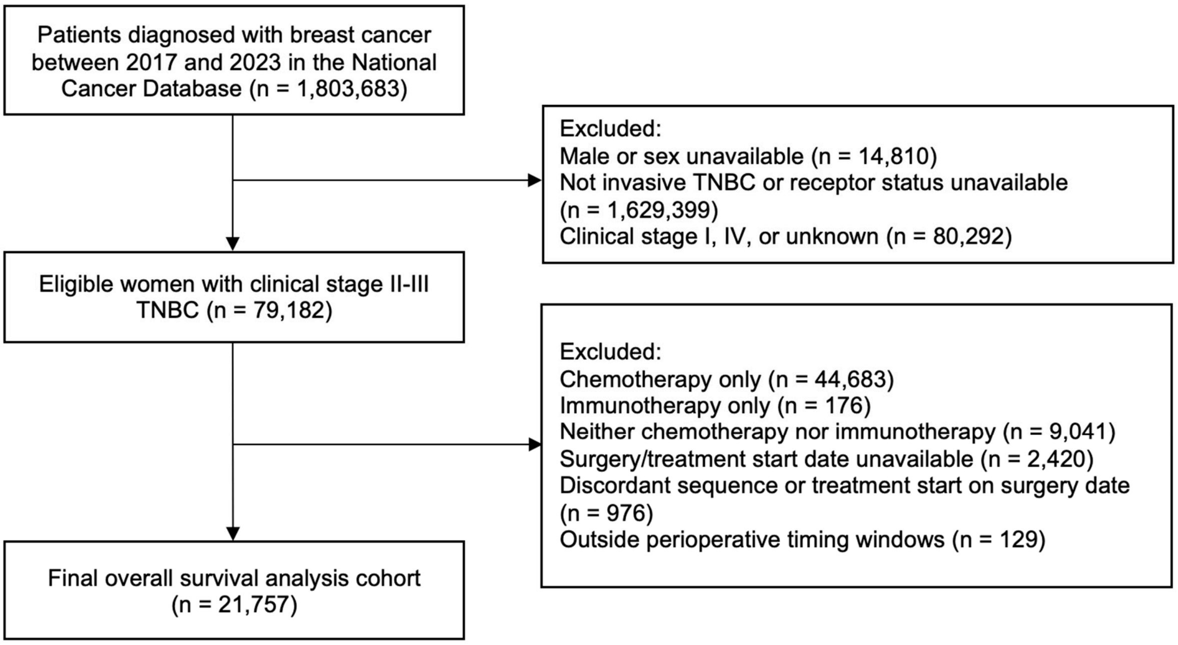

Abbreviations: TNBC, triple-negative breast cancer.

Supplementary Table S1. National Cancer Database variables and coding criteria used for patient selection and treatment classification.

Selection criterion	NCDB variable	Inclusion criteria / codes
Diagnosis year	YEAR_OF_DIAGNOSIS	2017–2023
Age	AGE	≥18 years
Sex	SEX	2 (female)
Invasive malignancy	BEHAVIOR	3 (invasive), when available
TNBC receptor status, 2017	CS_SITESPECIFIC_FACTOR_1 (ER)<br>CS_SITESPECIFIC_FACTOR_2 (PR)<br>CS_SITESPECIFIC_FACTOR_15 (HER2)	020 (negative) for all three receptors
TNBC receptor status, 2018–2023	ER_SUMMARY (ER)<br>PR_SUMMARY (PR)<br>HER2_OVERALL_SUMM (HER2)	0 or 00 (negative) for all three receptors
Clinical stage group	TNM_CLIN_STAGE_GROUP (2017)<br>AJCC_TNM_CLIN_STG_GRP (2018–2023)	Clinical stage II–III
Clinical T stage	TNM_CLIN_T (2017)<br>AJCC_TNM_CLIN_T (2018–2023)	cT1–cT4; excluded cT0 and unknown/unclassifiable values
Clinical N stage	TNM_CLIN_N (2017)<br>AJCC_TNM_CLIN_N (2018–2023)	cN0–cN3; excluded unknown/unclassifiable values
Clinical M stage	TNM_CLIN_M (2017)<br>AJCC_TNM_CLIN_M (2018–2023)	cM0
Chemotherapy receipt	RX_SUMM_CHEMO	01, 02, or 03 (also read as 1, 2, or 3)
Immunotherapy receipt	RX_SUMM_IMMUNOTHERAPY	01 (also read as 1)
Surgery timing	DX_DEFSURG_STARTED_DAYS; fallback:<br>DX_SURG_STARTED_DAYS	Valid recorded days from diagnosis to surgery
Chemotherapy initiation	DX_CHEMO_STARTED_DAYS	Valid recorded days from diagnosis to initiation
Immunotherapy initiation	DX_IMMUNO_STARTED_DAYS	Valid recorded days from diagnosis to initiation
Overall survival	DX_LASTCONTACT_DEATH_MONTHS<br>PUF_VITAL_STATUS	Positive survival duration (<9999 months); vital-status code 0 (deceased) or 1 (alive)

Abbreviations: NCDB, National Cancer Database; TNBC, triple-negative breast cancer.

Supplementary Table S2. Propensity-score model covariate balance before and after overlap weighting

		Original cohort			Overlap-weighted cohort		
Variable	Category	Neoadjuvant	Adjuvant	SMD	Neoadjuvant	Adjuvant	SMD
Patients		21,340	417		387.6	387.6	
Age, years		53.8 (13.3)	61.2 (14.9)	0.525	60.8 (13.5)	60.8 (14.9)	<0.001
Race/ethnicity	Non-Hispanic White	12,610 (59.1%)	267 (64.0%)	0.102	63.6%	63.6%	<0.001
	Non-Hispanic Black	4,606 (21.6%)	81 (19.4%)		19.6%	19.6%	
	Non-Hispanic Asian/Pacific Islander	1,099 (5.1%)	22 (5.3%)		5.4%	5.4%	
	Hispanic	2,317 (10.9%)	34 (8.2%)		8.3%	8.3%	
	Other/Unknown	708 (3.3%)	13 (3.1%)		3.1%	3.1%	
Insurance	Private	12,593 (59.0%)	175 (42.0%)	0.465	43.0%	43.0%	<0.001
	Medicare	4,988 (23.4%)	187 (44.8%)		43.7%	43.7%	
	Medicaid	2,760 (12.9%)	33 (7.9%)		8.0%	8.0%	
	Uninsured	455 (2.1%)	12 (2.9%)		2.9%	2.9%	
	Other/Unknown	544 (2.5%)	10 (2.4%)		2.4%	2.4%	
Charlson–Deyo comorbidity score	0	17,827 (83.5%)	320 (76.7%)	0.171	77.4%	77.4%	<0.001
	1	2,555 (12.0%)	67 (16.1%)		15.6%	15.6%	
	≥2	958 (4.5%)	30 (7.2%)		7.0%	7.0%	
Clinical T stage	cT1	1,799 (8.4%)	45 (10.8%)	0.165	10.8%	10.8%	<0.001
	cT2	14,477 (67.8%)	307 (73.6%)		73.1%	73.1%	
	cT3	3,509 (16.4%)	45 (10.8%)		11.0%	11.0%	
	cT4	1,555 (7.3%)	20 (4.8%)		5.1%	5.1%	
Clinical N stage	cN0	10,899 (51.1%)	288 (69.1%)	0.374	68.3%	68.3%	<0.001
	cN1	8,131 (38.1%)	110 (26.4%)		26.9%	26.9%	
	cN2-3	2,310 (10.8%)	19 (4.6%)		4.8%	4.8%	
Clinical grade	Grade 1-2	3,158 (14.8%)	86 (20.6%)	0.382	20.2%	20.2%	<0.001
	Grade 3	17,026 (79.8%)	262 (62.8%)		64.1%	64.1%	
	Unknown	1,156 (5.4%)	69 (16.5%)		15.7%	15.7%	
Histology	Ductal	19,974 (93.6%)	344 (82.5%)	0.347	83.5%	83.5%	<0.001
	Metaplastic	483 (2.3%)	41 (9.8%)		9.1%	9.1%	
	Other	883 (4.1%)	32 (7.7%)		7.4%	7.4%	
Diagnosis year	2017	188 (0.9%)	17 (4.1%)	0.358	3.7%	3.7%	<0.001
	2018	348 (1.6%)	41 (9.8%)		8.6%	8.6%	
	2019	408 (1.9%)	34 (8.2%)		7.3%	7.3%	
	2020	943 (4.4%)	34 (8.2%)		8.1%	8.1%	
	2021	3,995 (18.7%)	84 (20.1%)		20.7%	20.7%	
	2022	7,558 (35.4%)	107 (25.7%)		26.7%	26.7%	
	2023	7,900 (37.0%)	100 (24.0%)		25.0%	25.0%	
Facility type	Community Cancer Program	562 (2.6%)	30 (7.2%)	0.214	6.9%	6.9%	<0.001
	Comprehensive Community Cancer Program	5,442 (25.5%)	118 (28.3%)		28.1%	28.1%	
	Academic/Research Program	6,030 (28.3%)	109 (26.1%)		26.3%	26.3%	
	Integrated Network Cancer Program	5,726 (26.8%)	120 (28.8%)		28.6%	28.6%	
	Unknown	3,580 (16.8%)	40 (9.6%)		10.0%	10.0%	
Urban/rural residence	Metro	18,356 (86.0%)	341 (81.8%)	0.116	82.3%	82.3%	<0.001
	Urban	2,244 (10.5%)	59 (14.1%)		13.8%	13.8%	
	Rural/Unknown	740 (3.5%)	17 (4.1%)		3.9%	3.9%	

Abbreviation: SMD, standardized mean difference.

Supplementary Figure S2. Covariate balance before and after propensity-score overlap weighting

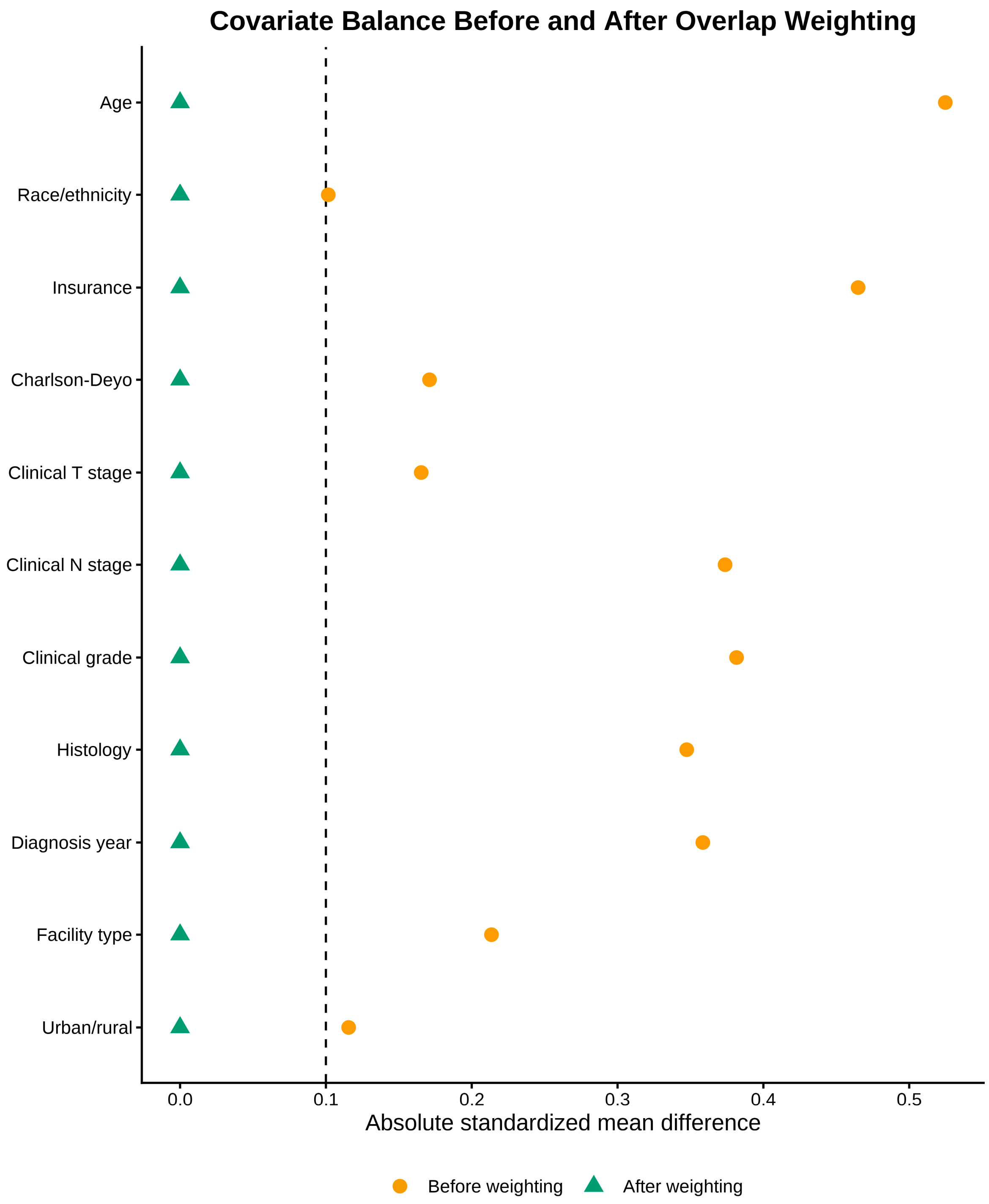

Supplementary Figure S3. Propensity-score distributions

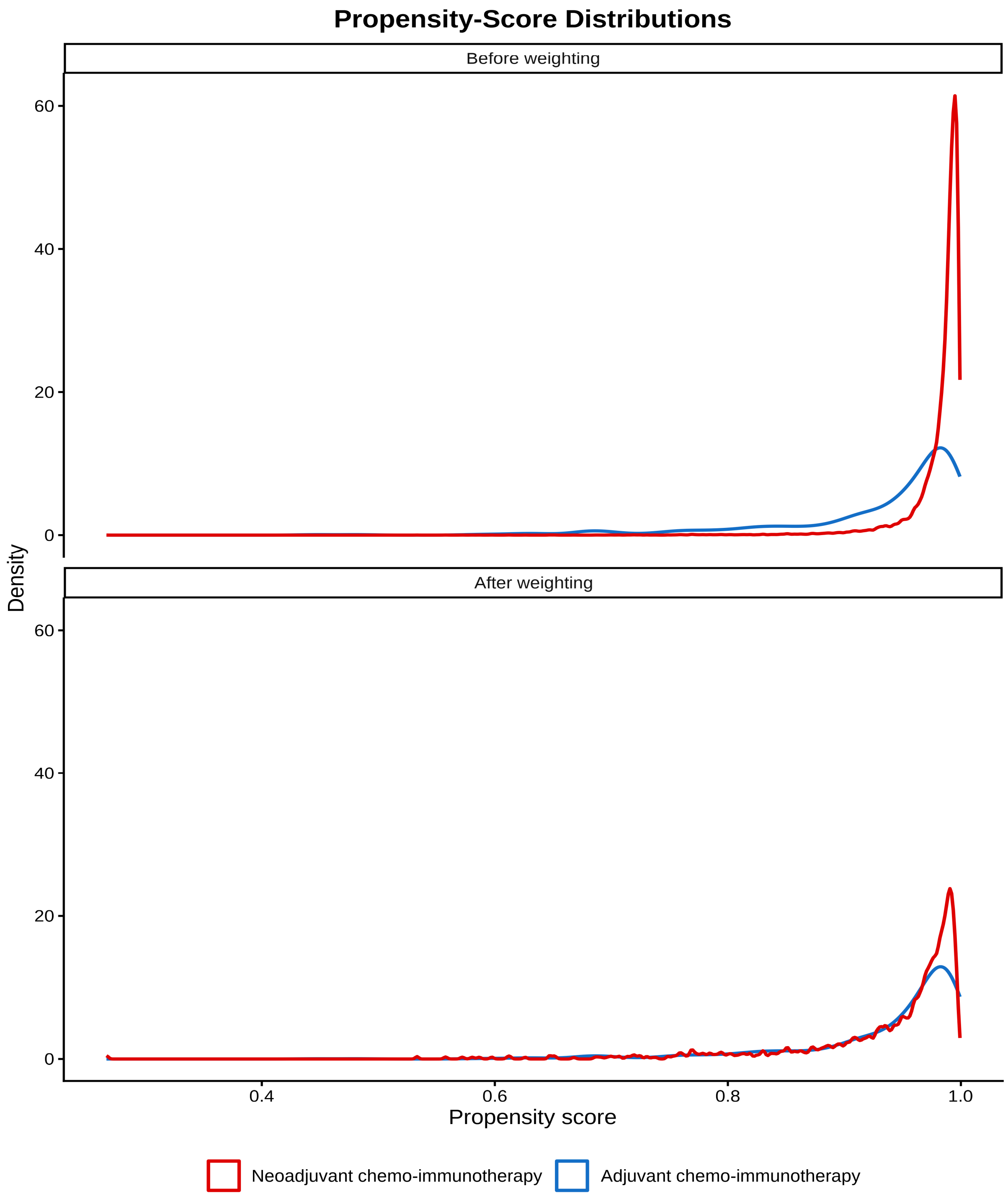

Supplement: Supplementary file 1 [file cancers-18-03490-s001.zip › cancers-4617513-supplementary.pdf]
